# ^18^ F-FDG PET Brain Findings in a Case of Idiopathic Benign Rolandic Epilepsy of Childhood

**DOI:** 10.1055/s-0042-1757287

**Published:** 2022-10-28

**Authors:** Kousik Vankadari, Rajender Kumar, Bhagwant Rai Mittal, Naveen Sankhyan

**Affiliations:** 1 Department of Nuclear Medicine, Yashoda Hospital, Secunderabad, Telangana, India; 2 Department of Nuclear Medicine, Postgraduate Institute of Medical Education and Research, Chandigarh, India; 3 Department of Pediatric Neurology, Postgraduate Institute of Medical Education and Research, Chandigarh, India

**Keywords:** ^18^ F-FDG, BCECTS, rolandic epilepsy, centrotemporal spikes, childhood epilepsy

## Abstract

Idiopathic benign rolandic epilepsy, also known as benign childhood epilepsy with centrotemporal spikes (BCECTS), is one of the commonly seen electroclinical epilepsy syndromes of childhood with a generally favorable long-term prognosis. We describe a 5-year-old female child who presented with recurrent focal seizures involving right side of face since the age of 6 months. She had no perinatal or postnatal insults, had normal development, and her neurological examination was unremarkable. Electroencephalogram showed rolandic spikes, suggesting BCETCS. Her seizures remained refractory to two appropriately dosed antiepileptic drugs. Magnetic resonance imaging of the brain did not reveal any structural lesion. Interictal fluorodeoxyglucose ^18^ F-positron emission tomography brain showed hypometabolism in the left lower rolandic region.

## Introduction

Benign rolandic epilepsy (BRE) is a commonly seen focal childhood epileptic disorder of probable genetic origin in view of family history of either febrile seizures or epilepsy in approximately 25% of pediatric children affected with this disorder. 1 It is named as rolandic epilepsy as seizures originate from cortex surrounding the central sulcus of brain that is called as centrotemporal area or rolandic area.

## Case Report

**Fig. 1:**
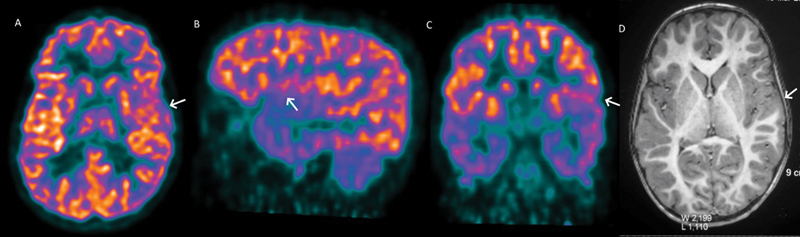
Interictal fluorodeoxyglucose ^18^ F-positron emission tomography images showing hypometabolism in the left lower rolandic motor cortex representing facial region ( **A** – **C** ; *arrows* ) with no significant morphological abnormality in the corresponding magnetic resonance imaging brain image ( **D** ; *arrow* ).

A 5-year-old female child presented with recurrent drug-resistant focal motor seizures involving right side of face with preserved awareness since age of 6 months. Initially she had 1 to 2 episodes/year that gradually increased to 4 to 5 episodes per week. Most of the seizures were early in the morning and were preceded by an aura of altered taste sensation and a feeling of the face being move to one side followed by clonic activity of right side of the face and drooling of saliva from the angle of the mouth associated with slurring of speech. The overt seizure episode lasts for approximately 1 minute with preserved consciousness. She was born at term and had a smooth perinatal transition. She was developmentally normal and did not suffer any postnatal brain insult. Her social behavior was age-appropriate and continued to show a good scholastic performance. Her intelligence quotient (by the Malin's Intelligence Scale for Indian Children) was 105 that was normal. Sleep electroencephalogram (EEG) revealed frequent bilateral (right > left) frontotemporal interictal epileptiform discharges with tangential dipoles (rolandic spikes) of benign childhood epilepsy with centrotemporal spikes (BCECTS). Since her seizures were refractory to maximal doses of two appropriate antiepileptic drugs (oxcarbazepine and levetiracetam), she was evaluated with radiological and function brain imaging to rule out a structural lesion amenable to surgical intervention. Fluorodeoxyglucose ^18^ F ( ^18^ F-FDG, ^148^ MBq) was intravenously administered to the patient. Thereafter child was allowed to rest in quiet, dim-lit room for 45 minute and static brain acquisition was done under positron emission tomography (PET) scanner. Transaxial, sagittal, and coronal interictal ^18^ F-FDG PET brain images done 72 hours following last seizure episode reveal hypometabolism in the left lower rolandic motor cortex representing facial region ( Fig. 1A – C ) with no structural abnormality in the corresponding region on transaxial magnetic resonance imaging (MRI) brain image ( Fig. 1D ). In addition to visual analysis, semiquantitative analysis was done by drawing an equal sized region of interest (ROI) on bilateral lower Rolandic cortices and maximum standardized uptake value (SUVmax) measured showed significant difference between right and left lower rolandic cortices with right to left asymmetry index measuring 18%.

## Discussion

BRE is a common focal idiopathic childhood epilepsy syndromes characterized by abnormal neuronal activity in the rolandic region of brain. 2 It is also referred to as benign childhood epilepsy with centrotemporal spikes (BCECTS) due to hallmark presence of rolandic spikes in centrotemporal region on EEG. 3 Seizures can start anywhere between 1 and 14 years (peak between 7 and 10 years), with an atypical presentation (earlier age of onset) seen in the index case. 4 The exact cause for epileptogenesis remains unknown, but literature suggests genetic disturbances in neurotransmission and delayed cortical maturation in the affected regions. 5 6 Most of the children usually have seizures in first few hours of sleep, but minority present with early morning seizures in wakefulness as seen in this case. Seizures associated with this syndrome are usually unilateral and manifestations include orofacial clonic movements, numbness/stiffness/tingling sensation of the face and throat, pharyngolaryngeal involvement, leading to guttural sounds, hypersalivation, and speech arrest or slurring of speech. 7 Occasionally facial numbness/twitching associated with the seizure disorder can spread to ipsilateral arm, ipsilateral leg, and to contralateral side, leading to full-blown generalized seizure. Most of the children affected with this disorder become seizure-free by the age of 15 to 16 years. 8 Even though children affected with this syndrome over the years have shown excellent long-term neurocognitive outcome, recent emerging studies report higher risk of developing subtle learning deficits and behavioral disorders. 9 10 Rarely BRE can be early manifestation of other epileptic syndromes. For lateralizing the seizure onset zone through interictal PET, concordance of visual asymmetric hypometabolism with electroclinical features plays important role. However, accuracy of visual interpretation can be further improved by deriving age-matched normalized Z score using statistical parametric mapping (SPM) and semiquantitative assessment of asymmetric hypometabolism using ROI-based SUVmax. Although SPM is a commonly used technique for the lateralization of seizures in adult patients, it is rarely applied to childhood studies due to the lack of validation of the spatial normalization procedure in children of different age groups. 11 Few studies reported in literature mentioned semiquantitative assessment of hypometabolism using SUVmax for seizure analysis and asymmetry index greater than 15% between affected and contralateral sides was considered to be significant. 12 To the best of our knowledge, this is the first case reporting interictal ^18^ F-FDG PET findings in BRE. The findings in our case suggest ^18^ F-FDG PET can be used as a complementary imaging modality to EEG, clinical features, and MRI brain to diagnose and confirm BRE in cases of discordant electroclinical and structural imaging results.
